# Obesity, Type 2 Diabetes and Bone in Adults

**DOI:** 10.1007/s00223-016-0229-0

**Published:** 2017-03-09

**Authors:** Jennifer S. Walsh, Tatiane Vilaca

**Affiliations:** Academic Unit of Bone Metabolism, Mellanby Centre for Bone Research, University of Sheffield, Sheffield, UK

**Keywords:** Bone, Obesity, Diabetes, Fat, Fracture

## Abstract

In an increasingly obese and ageing population, type 2 diabetes (T2DM) and osteoporotic fracture are major public health concerns. Understanding how obesity and type 2 diabetes modulate fracture risk is important to identify and treat people at risk of fracture. Additionally, the study of the mechanisms of action of obesity and T2DM on bone has already offered insights that may be applicable to osteoporosis in the general population. Most available evidence indicates lower risk of proximal femur and vertebral fracture in obese adults. However the risk of some fractures (proximal humerus, femur and ankle) is higher, and a significant number fractures occur in obese people. BMI is positively associated with BMD and the mechanisms of this association in vivo may include increased loading, adipokines such as leptin, and higher aromatase activity. However, some fat depots could have negative effects on bone; cytokines from visceral fat are pro-resorptive and high intramuscular fat content is associated with poorer muscle function, attenuating loading effects and increasing falls risk. T2DM is also associated with higher bone mineral density (BMD), but increased overall and hip fracture risk. There are some similarities between bone in obesity and T2DM, but T2DM seems to have additional harmful effects and emerging evidence suggests that glycation of collagen may be an important factor. Higher BMD but higher fracture risk presents challenges in fracture prediction in obesity and T2DM. Dual energy X-ray absorptiometry underestimates risk, standard clinical risk factors may not capture all relevant information, and risk is under-recognised by clinicians. However, the limited available evidence suggests that osteoporosis treatment does reduce fracture risk in obesity and T2DM with generally similar efficacy to other patients.

## Obesity, Type 2 Diabetes and Bone

Obesity is a major and growing public health problem; for example, in the UK, 40% of adults will be obese by 2025 [1]. Obesity is the most important risk factor for type 2 diabetes (T2DM), and the global prevalence of T2DM is likely to be 592 million by 2035 [2]. As the population ages, the burden of osteoporosis and fragility fracture also increases. Obesity and T2DM have effects on fracture risk, and fractures in T2DM are associated with greater morbidity than in the general population. Understanding how to assess and treat fracture risk in these groups is important for health care planning and individual patients. Additionally, the study of the mechanisms of action of obesity and T2DM on bone has already offered insights that may be applicable in the broader study of osteoporosis, such as the effects of adipokines on bone cells and the effects of collagen glycation on material properties of bone. There are some similarities in the effect of obesity and T2DM on bone, but some important differences such as cortical porosity and collagen glycation.

In this review, we describe the effects of obesity and T2DM on fracture risk and discuss possible mechanisms of their effects. We also consider the validity of existing fracture risk prediction tools and efficacy of osteoporosis treatment in these patient groups.

## Obesity, Fracture and BMD

Most of the available evidence supports a lower risk of proximal femur and vertebral fracture in obese adults [3]. However, fracture risk in obesity is not lower at all skeletal sites; the risk of some non-spine fractures including proximal humerus (RR 1.28), upper leg (OR 1.7) and ankle fracture (OR 1.5) is higher [4, 5]. A large number of low-trauma fractures occur in overweight and obese men and women, and the prevalence of low-trauma fractures is similar in obese and non-obese women [6]. Therefore, obesity is not entirely protective against fracture, and there are some site-specific effects on fracture.

There is a positive association between body mass index (BMI) and bone mineral density (BMD) [7]. BMD by dual-energy X-ray absorptiometry (DXA) is higher in obese people, but higher BMI and soft tissue thickness cause error in DXA measurement [8] through assumptions about abdominal thickness and beam hardening effects. However, other quantitative imaging methods (CT and ultrasound) also support higher BMD by DXA (although other methods are also subject to some influence from surrounding soft tissue). Calcaneus bone stiffness by ultrasound is greater in obesity [9] and by high-resolution peripheral quantitative computed tomography (HR-pQCT), obese adults have higher BMD, higher cortical BMD, higher trabecular BMD and greater trabecular number at the distal radius and distal tibia [10, 11]. Radius and tibia strength estimated by finite element analysis from HR-pQCT is greater in obesity than in normal weight controls [10]. Therefore, BMD probably is truly higher in obesity, and there is no site-specific BMD deficit to explain the site-specific fracture risk.

It is possible that even if BMD increases in response to obesity, the capacity for increase is limited and eventually the load-to-strength ratio rises far enough to cause fracture in low-trauma injuries. The increase in radius and tibia strength by HR-pQCT in obesity is proportionally less than the increase in BMI [11]. At the hip, by QCT and DXA, obese people have favourable features for bone strength, but the load-to-strength ratio is greater than normal weight controls [12, 13]. Greater soft-tissue thickness over the lateral hip dissipates fall impact, and so may continue to protect against hip fracture at high body weight even when load-to-strength ratio is exceeded [12, 14]. Intramuscular fat content is increased in obesity, and may be associated with poorer muscle function and increased fracture risk (‘dynapenic obesity’) [15, 16]. Poorer muscle function could increase falls and injury when falling, and there are data showing an excess of falls in obese people [17].

Thus, although BMD is higher in obesity, it may not be increased sufficiently to resist the greater forces acting when obese people fall. Non-bone factors such as muscle function and soft tissue thickness should also be considered as contributory and protective factors.

## Mechanisms of Action of Obesity on Bone

Some insight into how obesity may exert effects on bone can be obtained from biochemical markers of bone turnover. Biochemical markers are lower in obesity than in normal weight [18], but the difference in resorption markers may be greater than the difference in formation markers. This results in a higher uncoupling index in obesity, suggesting positive bone balance which helps to maintain bone mass in adulthood and with ageing [10]. Menopause causes a rapid increase in bone turnover, with net higher bone resorption and negative bone balance leading to bone loss. Higher body weight is associated with slower menopausal bone loss [19] consistent with a tendency towards positive bone balance in obesity.

One possible mechanism for higher BMD in obesity is increased mechanical loading and strain. Obese adults have increased body fat mass, but also increased lean mass, so passive loading and muscle-induced strain may have effects on bone modelling, density and geometry. However, impaired muscle function due to intramuscular fat accumulation could attenuate the positive effects of increased muscle mass on bone. If the dominant mechanism acting to increase BMD were physical loading, an increase in bone size by periosteal apposition might be expected. Hip cross-sectional area by DXA and QCT is increased in obesity [12, 13], but bone size at the radius and tibia by HR-pQCT does not differ between obese and normal weight controls [10]. Therefore, loading probably does not explain all of the action of obesity on bone.

Obesity has effects on a number of hormones known to act on bone, and so may act on bone through endocrine pathways. Adipocytes produce endocrine factors shown to influence bone cell number and activity. Leptin is produced by adipocytes, and circulating leptin levels reflect body fat mass with a primary role to regulate long-term energy balance by signalling satiety in the hypothalamus and reducing food intake. Circulating leptin acts on bone cells directly to increase bone formation [20], but when acting through the hypothalamus, it may inhibit bone formation through increased activation of the sympathetic nervous system [21]. The evidence from clinical human studies suggests that the dominant effect in vivo is probably the peripheral action to increase BMD [22]. Adiponectin is secreted in inverse proportion to fat mass, and has roles in the regulation of glucose and lipid metabolism. In humans, circulating adiponectin levels are inversely related to BMD [23]. Osteoclasts and osteoblasts express adiponectin receptors, and there is some experimental evidence that adiponectin could modulate RANK/RANK-ligand/OPG signalling [24]. Similarly to leptin, mouse studies suggest that adiponectin may also signal through the central nervous system to regulate bone turnover through autonomic innervation [25]. However, the dominant mechanism through which it acts on the skeleton in obese humans in vivo is not yet clear. Adipocytes express aromatase, and aromatisation of androgens is the main source of oestrogen in postmenopausal women and men. High fat mass is associated with higher circulating estradiol, and so aromatase activity is likely to contribute to positive effects of fat on bone, particularly in postmenopausal women [26].

Pancreatic and gut hormone secretion is altered in obesity and may influence bone metabolism. Insulin, amylin and preptin are increased in obesity, and may have direct effects on bone cells to increase bone formation and decrease resorption. Insulin may also have indirect positive effects on bone by decreasing hepatic sex-hormone binding globulin production, increasing bioavailability of oestrogen and androgens. Ghrelin, gastric inhibitory polypeptide (GIP) and glucagon-like peptide 2 (GLP-2) have direct and indirect effects on bone metabolism, driven by the acute response to food intake and more long-term energy balance [27].

Serum 25-hydroxy vitamin D (25OHD) is lower in obesity than normal weight controls, but this is likely to reflect greater volume of distribution (into fat, muscle and extracellular fluid). Therefore, serum 25OHD may not indicate low whole-body vitamin D status in obesity, and it does not seem to be associated with lower BMD or higher bone turnover. It is possible that the lower vitamin D in obesity would adversely affect BMD, but that the other positive effects of obesity on BMD are dominant [28].

**Fig. 1:**
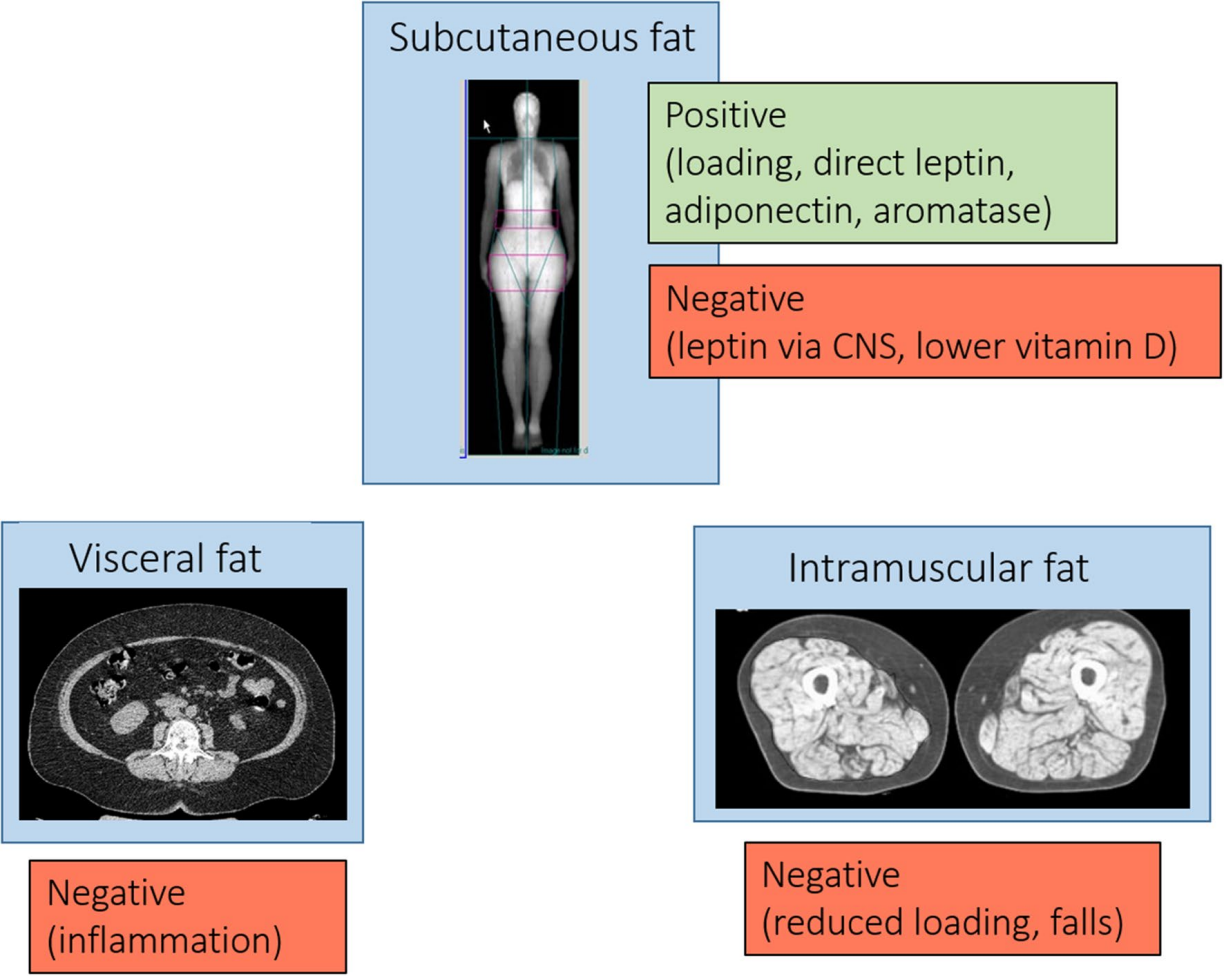
Fat depot actions on bone in obesity

Not all fat is the same, and some fat depots could have negative effects on bone (Fig. 1). Subcutaneous and visceral fat have different metabolic profiles, and pro-inflammatory cytokines from visceral fat such as interleukin-6 (IL-6) and tumour necrosis factor alpha (TNF-α) increase bone resorption, so may have harmful effects on BMD [29]. In support of adverse actions, greater central and visceral adiposity is associated with lower BMD and some adverse microstructural features from bone biopsy and HR-pQCT but the relationship may vary with age and gender [30–32].

## Fracture Risk Assessment and Osteoporosis Treatment in Obesity

Fracture risk assessment in clinical practice uses bone densitometry by DXA and clinical risk factors. This offers some challenges in obesity—the precision of DXA measurements is reduced in obesity due to effects of soft tissue thickness [8]. Also, because fracture pattern differs between obese and normal weight groups, and we do not yet fully understand the cause of fractures in obesity, the usual fracture prediction tools might not be expected to perform so well. However, FRAX (with and without BMD) predicted hip and major osteoporotic fracture with similar accuracy in obese and non-obese postmenopausal women in the Study of Osteoporotic Fractures [33].

Most currently used drugs for osteoporosis are anti-resorptive. Because bone turnover and bone resorption are already reduced in obesity, the question has been raised whether anti-resorptive treatment is effective for fracture prevention in obesity. The key clinical trials of bisphosphonates did not include large numbers of obese people, but there are some available data. In the Horizon trial, 3 years of zoledronic acid decreased vertebral fracture more in postmenopausal women with BMI above 25 kg/m^2^ than women with BMI below 25 kg/m^2^ [34]. Non-vertebral fracture reduction did not differ by BMI. In the Freedom trial, with 3 years of denosumab, vertebral fracture risk reduction was independent of BMI, but non-vertebral fracture reduction was not significant in women with BMI above 25 kg/m^2^ [35].

## Type 2 Diabetes, Fracture and BMD

A number of meta-analyses have reported an increase in the risk of fractures in type 2 diabetes (T2DM) [36–40]. There is a 1.3- to 2.1-fold increased risk of hip fracture [36, 37, 39, 40] and 1.2-fold increased risk of other fractures [36, 37], but vertebral fracture risk does not seem to be increased [37, 40] (Table 1). The size of the fracture risk increase may be modest, but it is important to recognise that after fracture, patients with diabetes have greater mortality, develop more complications (such as renal impairment and cardiac problems) and recover less well than non-diabetic patients [41, 42].

**Table 1:** Risk of hip, spine and other any fractures in T2DM according to meta-analyses

Study	Hip fractures	Spine fractures	Other fractures
Janghorbani [32]	1.7 (1.3–2.2)*	1.2 (0.7–2.2)	Any fracture 1.2 (1.01–1.5)*
Vestergaard [33]	1.38 (1.25–1.53)*	0.93 (0.63–1.37)	Any fractures 1.19 (1.11–1.27)*
Fan [35]	2.07 (1.83–2.33)*	–	–
Dytfeld [36]	1.26 (1.07–1.57)*	1.13 (0.94–1.37)	–

*Statistically significant increase in the risk

Although fracture risk is higher, BMD is increased in T2DM (lumbar spine *Z*-score + 0.41, total hip *Z*-score + 0.27) [37]. Nearly all people with T2DM are obese, and so the higher BMD in T2DM is probably due to similar mechanisms as those acting in non-diabetic obese people. In addition, high circulating insulin could increase osteoblast activity and bone formation [43]. The increase in foot and ankle fractures is consistent with the pattern seen in obesity, but the increase in hip fracture risk is discrepant between T2DM and non-diabetic obese, so additional factors may be acting to increase bone fragility in T2DM.

Microarchitecture studies with HR-pQCT suggest a cortical strength deficit in T2DM. There is decreased cortical thickness and volumetric BMD (vBMD), with increased cortical porosity and pore size in T2DM [44] patients with microvascular disease (retinopathy, neuropathy or nephropathy). These changes are associated with decreased bone strength by finite element analysis [44, 45] and are greater in T2DM patients with previous fractures [46], suggesting that they may be clinically significant contributors to fracture risk.

## Mechanisms of Action of T2DM on Bone

Bone turnover markers studies in diabetes have had some conflicting results, but the most consistent finding is that markers of resorption (C-terminal cross-linking telopeptide of type I collagen (CTX), N-terminal cross-linking telopeptide of type I collagen (NTX)) and formation (procollagen type I N propeptide (P1NP), osteocalcin) are reduced [47, 48]. Methodological studies have excluded a direct effect of glucose in the measurements [48], so the bone turnover markers probably do reflect a true biological effect. Histomorphometry in T2D shows decrease in bone volume, osteoid volume, thickness and osteoblast surface, and poor uptake of label indicating reduced bone formation [49, 50] consistent with lower turnover from biochemical markers.

One important factor which may contribute to bone fragility in T2DM is post-translational glycation of collagen in bone matrix. Enzymatic cross-linking of collagen maintains the strength of normal bone matrix because the collagen matrix confers toughness, allowing the bone to endure plastic strain without breaking. Increasing numbers of cross-links reduces the plasticity of the matrix, and the bone breaks at lower strain. Older collagen has more cross-links and less plasticity. Exposure to high glucose levels promotes glycation of proteins [advanced glycation end products (AGEs)]. In collagen, AGEs lead to non-enzymatic cross-linking [51], and so could decrease plasticity and bone material strength [52]. Higher urine pentosidine (a marker of AGEs) is associated with higher vertebral fracture risk in post-menopausal non-diabetic women [53].

Bone material strength can be evaluated in vivo by reference point indentation (Osteoprobe). By this method, material strength is 10% lower in T2DM than matched controls. The difference persists after correction for BMI and is correlated with average HbA1c [54]. Indirect measurement of AGEs using skin auto fluorescence explains 26% of the reduced bone strength by indentation, and is associated with lower P1NP in patients with T2D [55]. Therefore, there is some evidence for an association of glucose exposure with poor bone material quality in T2DM, and collagen glycation is a plausible contributor to increased fracture risk.

Particularly for foot and ankle fracture, it is possible that neuropathy and vasculopathy in T2DM could have effects on bone cell function or bone material properties. Sympthetic tone contributes to the regulation of bone turnover, and the extreme example of Charcot foot demonstrates the potential for bone to dysfunction when normal sensory and autonomic innervation is lost. However, these factors have not yet been investigated in the context of diabetes and fracture. Besides the intrinsic bone properties, other factors could increase the risk of fractures in T2DM. Poor metabolic control, hypoglycaemia and neuropathy increase falls risk [56, 57], and in meta-analysis, hypoglycaemia was associated with fracture (OR 1.92) [58]. However, increased fracture risk persists after correction for falls [59].

Diabetes treatment may also modulate fracture risk [60]. Metformin and sulfonylureas have neutral or slightly protective associations with fracture risk [60, 61]. It is possible that metformin increases osteoblast activity through Runt-related transcription factor 2 (Runx2) signalling. Thiazolidinediones (TZDs, glitazones) activate peroxisome proliferator-activated receptor gamma (PPARγ) which decreases insulin resistance. Activation of PPARγ suppresses IGF-1 expression in bone and drives differentiation of mesenchymal stem cells to adipocytes rather than to osteoblasts [62]. TZD use is associated with increased fracture risk—the ADOPT study reported cumulative incidence of fractures of 15.1% with rosiglitazone versus 7.3% with metformin [63]. Sodium–glucose cotransporter-2 (SGLT2) inhibitors have been associated with increased fracture risk [64], possibly through increased renal phosphate reabsorption leading to increased parathyroid hormone (PTH) and increased bone resorption [65]. Gut peptides such as GLP-2 decrease turnover in response to feeding, and there has been interest in the possible bone effects of GLP-1 analogues and dipeptidyl peptidase 4 (DPP-4) inhibitors in diabetes treatment. So far, there is no clear evidence of an effect on fracture risk [66]. Fracture risk is higher in people treated with insulin than with oral agents, but this may reflect insulin use as a marker of longer duration of disease, poorer control and more microvascular complications rather than a direct biological effect.

## Fracture Risk Assessment and Osteoporosis Treatment in T2DM

Because BMD is increased in T2DM, and there are disease-specific risk factors such as microvascular disease and collagen glycation, standard fracture risk assessments using DXA and clinical risk factors (including FRAX) underestimate fracture risk in T2DM [67]. Inadequate or inaccurate risk assessment is reflected in the observation that people with diabetes are less likely to be prescribed bisphosphonates than those without diabetes [68]. This may be partly due to underestimation of risk by assessment tools, but also because clinicians do not recognise that people with diabetes are at risk of fracture, so do not assess their risk or treat.

Although the pathophysiology of fracture in T2DM differs from postmenopausal osteoporosis, (particularly in that bone turnover is low in T2DM), osteoporosis treatments do reduce fracture in T2DM. In post hoc analysis of the FIT alendronate and HORIZON zoledronic acid trial, fracture reduction was similar in participants with and without diabetes [69]. Teriparatide and sclerostin antibodies increase BMD in Zucker diabetic rats, but the rats’ bone phenotype is different from human T2DM, and there is not yet available information on these drugs in humans with T2DM [70, 71].

## Summary and Discussion

Obesity in adults is protective against some fractures, particularly hip fractures. However, some fractures, such as ankle and humerus are more common in obesity, and the prevalence of low-trauma fractures is similar in obese and non-obese women. BMD in obese people is higher at all sites, bone turnover is lower, and bone strength measures suggest that obesity is favourable for bone strength, but bone strength does not seem sufficiently increased to protect against all fractures. Therefore, explanations for the fracture pattern in obesity need to consider other factors such as load-to-strength ratio, soft tissue padding, muscle function and falls. Finite element models incorporating patient-specific factors such as height, weight, soft tissue thickness and quantitative gait assessment with bone physical measurements may offer a route of investigation for these potential contributors.

There are many possible mechanisms acting on bone metabolism in obesity, such as adipokines and gut hormones. Some of these are potential therapeutic targets for the treatment of osteoporosis in obese and non-obese people.

Type 2 diabetes is associated with increased BMD and lower bone turnover but increased overall risk of fracture and hip fracture. Some of the mechanisms acting to increase BMD in obesity are likely to be relevant in T2DM, but the pathophysiology of bone fragility in T2DM is not yet clearly understood. Additional influence of AGEs on bone matrix and complications of diabetes are likely to contribute to the increased fracture risk, and AGE markers might be of value in further research and clinical assessment. If glucose exposure and diabetes complications are major contributors, the most effective strategy to reduce fracture risk may be to improve glycaemic control. Fracture risk in T2DM is under-recognised, under-estimated and undertreated, but anti-resorptives do seem to be effective in fracture prevention. If diabetes bone disease is a low-turnover state, it would be interesting to see whether it responds well to anabolic bone agents or whether the underlying pathology impairs the anabolic response.

As our populations become older and more obese, understanding the interactions of obesity, T2DM and fracture is becoming a pressing need to reduce the societal and individual costs of fracture.

## References

[1] ButlandJebb BS, Kopelman P, McPherson K, Thomas S, Mardell J, Parry V (2007). Tackling obesities: future choices—Project Report.

[2] http://www.diabetes.org.uk/Documents/About%20Us/Statistics/Diabetes-key-stats-guidelines-April2014.pdf. Accessed Nov 2016

[3] De LC, Kanis JA, Oden A, Johanson H, Johnell O, Delmas P, Eisman JA, Kroger H, Fujiwara S, Garnero P, McCloskey EV, Mellstrom D, Melton LJ, Meunier PJ, Pols HA, Reeve J, Silman A, Tenenhouse A (2005). Body mass index as a predictor of fracture risk: a meta-analysis. Osteoporos Int.

[4] Compston JE, Watts NB, Chapurlat R, Cooper C, Boonen S, Greenspan S, Pfeilschifter J, Silverman S, Diez-Perez A, Lindsay R, Saag KG, Netelenbos JC, Gehlbach S, Hooven FH, Flahive J, Adachi JD, Rossini M, LaCroix AZ, Roux C, Sambrook PN, Siris ES (2011). Obesity is not protective against fracture in postmenopausal women: GLOW. Am J Med.

[5] Prieto-Alhambra D, Premaor MO, Fina Aviles F, Hermosilla E, Martinez-Laguna D, Carbonell-Abella C, Nogues X, Compston JE, Diez-Perez A (2012). The association between fracture and obesity is site-dependent: a population-based study in postmenopausal women. J Bone Miner Res.

[6] Premaor MO, Comim FV, Compston JE (2014). Obesity and fractures. Arq Bras Endocrinol Metabol.

[7] Yang S, Shen X (2015). Association and relative importance of multiple obesity measures with bone mineral density: the National Health and Nutrition Examination Survey 2005–2006. Arch Osteoporos.

[8] Knapp KM, Welsman JR, Hopkins SJ, Fogelman I, Blake GM (2012). Obesity increases precision errors in dual-energy X-ray absorptiometry measurements. J Clin Densitom.

[9] Berg RM, Wallaschofski H, Nauck M, Rettig R, Markus MR, Laqua R, Friedrich N, Hannemann A (2015). Positive association between adipose tissue and bone stiffness. Calcif Tissue Int.

[10] Evans AL, Paggiosi MA, Eastell R, Walsh JS (2015). Bone density, microstructure and strength in obese and normal weight men and women in younger and older adulthood. J Bone Miner Res.

[11] Sornay-Rendu E, Boutroy S, Vilayphiou N, Claustrat B, Chapurlat RD (2013). In obese postmenopausal women, bone microarchitecture and strength are not commensurate to greater body weight: the Os des Femmes de Lyon (OFELY) study. J Bone Miner Res.

[12] Bachmann KN, Fazeli PK, Lawson EA, Russell BM, Riccio AD, Meenaghan E, Gerweck AV, Eddy K, Holmes T, Goldstein M, Weigel T, Ebrahimi S, Mickley D, Gleysteen S, Bredella MA, Klibanski A, Miller KK (2014). Comparison of hip geometry, strength, and estimated fracture risk in women with anorexia nervosa and overweight/obese women. J Clin Endocrinol Metab.

[13] Shen J, Nielson CM, Marshall LM, Lee DC, Keaveny TM, Orwoll ES, Osteoporotic Fractures in Men Mr OSRG (2015). The association between BMI and QCT-derived proximal hip structure and strength in older men: a cross-sectional study. J Bone Miner Res.

[14] Majumder S, Roychowdhury A, Pal S (2008). Effects of trochanteric soft tissue thickness and hip impact velocity on hip fracture in sideways fall through 3D finite element simulations. J Biomech.

[15] Lang T, Cauley JA, Tylavsky F, Bauer D, Cummings S, Harris TB, Health ABCS (2010). Computed tomographic measurements of thigh muscle cross-sectional area and attenuation coefficient predict hip fracture: the health, aging, and body composition study. J Bone Miner Res.

[16] Scott D, Daly RM, Sanders KM, Ebeling PR (2015). Fall and fracture risk in sarcopenia and dynapenia with and without obesity: the role of lifestyle interventions. Curr Osteoporos Rep.

[17] Himes CL, Reynolds SL (2012). Effect of obesity on falls, injury, and disability. J Am Geriatr Soc.

[18] Garnero P, Sornay-Rendu E, Claustrat B, Delmas PD (2000). Biochemical markers of bone turnover, endogenous hormones and the risk of fractures in postmenopausal women: the OFELY study. J Bone Miner Res.

[19] Reid IR, Ames RW, Evans MC, Sharpe SJ, Gamble GD (1994). Determinants of the rate of bone loss in normal postmenopausal women. J Clin Endocrinol Metab.

[20] Cornish J, Callon KE, Bava U, Lin C, Naot D, Hill BL, Grey AB, Broom N, Myers DE, Nicholson GC, Reid IR (2002). Leptin directly regulates bone cell function in vitro and reduces bone fragility in vivo. J Endocrinol.

[21] Hamrick MW, Ferrari SL (2008). Leptin and the sympathetic connection of fat to bone. Osteoporos Int.

[22] Reid IR (2010). Fat and bone. Arch Biochem Biophys.

[23] Lenchik L, Register TC, Hsu FC, Lohman K, Nicklas BJ, Freedman BI, Langefeld CD, Carr JJ, Bowden DW (2003). Adiponectin as a novel determinant of bone mineral density and visceral fat. Bone.

[24] Luo XH, Guo LJ, Xie H, Yuan LQ, Wu XP, Zhou HD, Liao EY (2006). Adiponectin stimulates RANKL and inhibits OPG expression in human osteoblasts through the MAPK signaling pathway. J Bone Miner Res.

[25] Kajimura D, Lee HW, Riley KJ, Arteaga-Solis E, Ferron M, Zhou B, Clarke CJ, Hannun YA, DePinho RA, Guo XE, Mann JJ, Karsenty G (2013). Adiponectin regulates bone mass via opposite central and peripheral mechanisms through FoxO1. Cell Metab.

[26] Riis BJ, Rodbro P, Christiansen C (1986). The role of serum concentrations of sex steroids and bone turnover in the development and occurrence of postmenopausal osteoporosis. Calcif Tissue Int.

[27] Walsh JS, Henriksen DB (2010). Feeding and bone. Arch Biochem Biophys.

[28] Walsh JS, Evans AL, Bowles S, Naylor KE, Jones KS, Schoenmakers I, Jacques RM, Eastell R (2016). Free 25-hydroxyvitamin D is low in obesity, but there are no adverse associations with bone health. Am J Clin Nutr.

[29] Morley JE, Baumgartner RN (2004). Cytokine-related aging process. J Gerontol A Biol Sci Med Sci.

[30] Cohen A, Dempster DW, Recker RR, Lappe JM, Zhou H, Zwahlen A, Muller R, Zhao B, Guo X, Lang T, Saeed I, Liu XS, Guo XE, Cremers S, Rosen CJ, Stein EM, Nickolas TL, McMahon DJ, Young P, Shane E (2013). Abdominal fat is associated with lower bone formation and inferior bone quality in healthy premenopausal women: a transiliac bone biopsy study. J Clin Endocrinol Metab.

[31] Ng AC, Melton LJ, Atkinson EJ, Achenbach SJ, Holets MF, Peterson JM, Khosla S, Drake MT (2013). Relationship of adiposity to bone volumetric density and microstructure in men and women across the adult lifespan. Bone.

[32] Zhang P, Peterson M, Su GL, Wang SC (2015). Visceral adiposity is negatively associated with bone density and muscle attenuation. Am J Clin Nutr.

[33] Premaor M, Parker RA, Cummings S, Ensrud K, Cauley JA, Lui LY, Hillier T, Compston J, Study of Osteoporotic Fractures Research G (2013). Predictive value of FRAX for fracture in obese older women. J Bone Miner Res.

[34] Eastell R, Black DM, Boonen S, Adami S, Felsenberg D, Lippuner K, Cummings SR, Delmas PD, Palermo L, Mesenbrink P, Cauley JA, Trial HPF (2009). Effect of once-yearly zoledronic acid five milligrams on fracture risk and change in femoral neck bone mineral density. J Clin Endocrinol Metab.

[35] McClung MR, Boonen S, Torring O, Roux C, Rizzoli R, Bone HG, Benhamou CL, Lems WF, Minisola S, Halse J, Hoeck HC, Eastell R, Wang A, Siddhanti S, Cummings SR (2012). Effect of denosumab treatment on the risk of fractures in subgroups of women with postmenopausal osteoporosis. J Bone Miner Res.

[36] Janghorbani M, Van Dam RM, Willett WC, Hu FB (2007). Systematic review of type 1 and type 2 diabetes mellitus and risk of fracture. Am J Epidemiol.

[37] Vestergaard P (2007). Discrepancies in bone mineral density and fracture risk in patients with type 1 and type 2 diabetes—a meta-analysis. Osteoporosis Int.

[38] Shah VN, Shah CS, Snell-Bergeon JK (2015). Type 1 diabetes and risk of fracture: meta-analysis and review of the literature. Diabet Med.

[39] Fan Y, Wei F, Lang Y, Liu Y (2016). Diabetes mellitus and risk of hip fractures: a meta-analysis. Osteoporos Int.

[40] Dytfeld J, Michalak M (2016). Type 2 diabetes and risk of low-energy fractures in postmenopausal women: meta-analysis of observational studies. Aging Clin Exp Res.

[41] Hu F, Jiang C, Shen J, Tang P, Wang Y (2012). Preoperative predictors for mortality following hip fracture surgery: a systematic review and meta-analysis. Injury.

[42] Ekstrom W, Al-Ani AN, Saaf M, Cederholm T, Ponzer S, Hedstrom M (2013). Health related quality of life, reoperation rate and function in patients with diabetes mellitus and hip fracture—a 2year follow-up study. Injury.

[43] Zhang W, Shen X, Wan C, Zhao Q, Zhang L, Zhou Q, Deng L (2012). Effects of insulin and insulin-like growth factor 1 on osteoblast proliferation and differentiation: differential signalling via Akt and ERK. Cell Biochem Funct.

[44] Shanbhogue VV, Hansen S, Frost M, Jorgensen NR, Hermann AP, Henriksen JE, Brixen K (2016). Compromised cortical bone compartment in type 2 diabetes mellitus patients with microvascular disease. Eur J Endocrinol.

[45] Burghardt AJ, Issever AS, Schwartz AV, Davis KA, Masharani U, Majumdar S, Link TM (2010). High-resolution peripheral quantitative computed tomographic imaging of cortical and trabecular bone microarchitecture in patients with type 2 diabetes mellitus. J Clin Endocrinol Metab.

[46] Patsch JM, Burghardt AJ, Yap SP, Baum T, Schwartz AV, Joseph GB, Link TM (2013). Increased cortical porosity in type 2 diabetic postmenopausal women with fragility fractures. J Bone Miner Res.

[47] Starup-Linde J, Vestergaard P (2016). Biochemical bone turnover markers in diabetes mellitus—a systematic review. Bone.

[48] Starup-Linde J, Eriksen SA, Lykkeboe S, Handberg A, Vestergaard P (2014). Biochemical markers of bone turnover in diabetes patients—a meta-analysis, and a methodological study on the effects of glucose on bone markers. Osteoporos Int.

[49] Leite Duarte ME, da Silva RD (1996). Histomorphometric analysis of the bone tissue in patients with non-insulin-dependent diabetes (DMNID). Rev Hosp Clin Fac Med Sao Paulo.

[50] Manavalan JS, Cremers S, Dempster DW, Zhou H, Dworakowski E, Kode A, Kousteni S, Rubin MR (2012). Circulating osteogenic precursor cells in type 2 diabetes mellitus. J Clin Endocrinol Metab.

[51] Saito M, Fujii K, Mori Y, Marumo K (2006). Role of collagen enzymatic and glycation induced cross-links as a determinant of bone quality in spontaneously diabetic WBN/Kob rats. Osteoporos Int.

[52] Avery NC, Bailey AJ (2006). The effects of the Maillard reaction on the physical properties and cell interactions of collagen. Pathol Biol (Paris).

[53] Shiraki M, Kuroda T, Tanaka S, Saito M, Fukunaga M, Nakamura T (2008). Nonenzymatic collagen cross-links induced by glycoxidation (pentosidine) predicts vertebral fractures. J Bone Miner Metab.

[54] Farr JN, Khosla S (2016). Determinants of bone strength and quality in diabetes mellitus in humans. Bone.

[55] Furst JR, Bandeira LC, Fan WW, Agarwal S, Nishiyama KK, McMahon DJ, Dworakowski E, Jiang H, Silverberg SJ, Rubin MR (2016). Advanced glycation endproducts and bone material strength in type 2 diabetes. J Clin Endocrinol Metab.

[56] Tilling LM, Darawil K, Britton M (2006). Falls as a complication of diabetes mellitus in older people. J Diabetes Complicat.

[57] Hewston P, Deshpande N (2016). Falls and balance impairments in older adults with type 2 diabetes: thinking beyond diabetic peripheral neuropathy. Can J Diabetes..

[58] Mattishent K, Loke YK (2016). Meta-analysis: association between hypoglycaemia and serious adverse events in older patients. J Diabetes Complicat..

[59] Schwartz AV, Sellmeyer DE, Ensrud KE, Cauley JA, Tabor HK, Schreiner PJ, Jamal SA, Black DM, Cummings SR, Study of Osteoporotic Features Research G (2001). Older women with diabetes have an increased risk of fracture: a prospective study. J Clin Endocrinol Metab.

[60] Palermo A, D’Onofrio L, Eastell R, Schwartz AV, Pozzilli P, Napoli N (2015). Oral anti-diabetic drugs and fracture risk, cut to the bone: safe or dangerous? A narrative review. Osteoporos Int.

[61] Mannucci E, Dicembrini I (2015). Drugs for type 2 diabetes: role in the regulation of bone metabolism. Clin Cases Miner Bone Metab.

[62] Ali AA, Weinstein RS, Stewart SA, Parfitt AM, Manolagas SC, Jilka RL (2005). Rosiglitazone causes bone loss in mice by suppressing osteoblast differentiation and bone formation. Endocrinology.

[63] Kahn SE, Zinman B, Lachin JM, Haffner SM, Herman WH, Holman RR, Kravitz BG, Yu D, Heise MA, Aftring RP, Viberti G, Diabetes Outcome Progression Trial Study Group (2008). Rosiglitazone-associated fractures in type 2 diabetes: an analysis from a diabetes outcome progression trial (ADOPT). Diabetes Care.

[64] Watts NB, Bilezikian JP, Usiskin K, Edwards R, Desai M, Law G, Meininger G (2016). Effects of canagliflozin on fracture risk in patients with type 2 diabetes mellitus. J Clin Endocrinol Metab.

[65] Taylor SI, Blau JE, Rother KI (2015). Possible adverse effects of SGLT2 inhibitors on bone. Lancet Diabetes Endocrinol.

[66] Su B, Sheng H, Zhang M, Bu L, Yang P, Li L, Li F, Sheng C, Han Y, Qu S, Wang J (2015). Risk of bone fractures associated with glucagon-like peptide-1 receptor agonists’ treatment: a meta-analysis of randomized controlled trials. Endocrine.

[67] Giangregorio LM, Leslie WD, Lix LM, Johansson H, Oden A, McCloskey E, Kanis JA (2012). FRAX underestimates fracture risk in patients with diabetes. J Bone Miner Res.

[68] Fraser LA, Papaioannou A, Adachi JD, Ma J, Thabane L (2014). Fractures are increased and bisphosphonate use decreased in individuals with insulin-dependent diabetes: a 10year cohort study. BMC Musculoskelet Disord.

[69] Schwartz A, Vittinghof E, Bauer DC, Cummings SR, Grey A, McClung MR, Napoli N, Reid IR, Schafer AL, Wallace RB, Black DM (2015) Bisphosphonates reduce fracture risk in postmenopausal women with diabetes: Results from FIT and HORIZON trials. Paper presented at the American Society for Bone and Mineral Research

[70] Hamann C, Rauner M, Hohna Y, Bernhardt R, Mettelsiefen J, Goettsch C, Gunther KP, Stolina M, Han CY, Asuncion FJ, Ominsky MS, Hofbauer LC (2013). Sclerostin antibody treatment improves bone mass, bone strength, and bone defect regeneration in rats with type 2 diabetes mellitus. J Bone Miner Res.

[71] Hamann C, Picke AK, Campbell GM, Balyura M, Rauner M, Bernhardt R, Huber G, Morlock MM, Gunther KP, Bornstein SR, Gluer CC, Ludwig B, Hofbauer LC (2014). Effects of parathyroid hormone on bone mass, bone strength, and bone regeneration in male rats with type 2 diabetes mellitus. Endocrinology.

